# Possible Triggering of Molecular Mimicry by Environmental Pollution

**DOI:** 10.3390/biology15120944

**Published:** 2026-06-17

**Authors:** Sofya Mishina, Bryttan Adams, Bruce Uhal, Yun Liang

**Affiliations:** 1 Department of Physiology, Michigan State University, East Lansing, MI 48824, USA; 2 Department of Pharmacology and Toxicology, Michigan State University, East Lansing, MI 48824, USA

**Keywords:** autoimmune diseases, molecular mimicry, infection, environmental pollution, benzo[a]pyrene

## Abstract

The ‘molecular mimicry theory’ states that foreign antigens introduced during infection can activate autoreactive T or B cells, leading to autoimmune diseases. However, despite a decrease in infectious burden, autoimmune disease incidence is on the rise globally. To address this paradox, we analyzed the impact of benzo[a]pyrene (BaP) on the autoimmune-prone mouse strain MRL. We found that BaP induced the expression of genes, the protein products of which showed homology to bacterial pathogens, in the MRL spleen and kidneys. Based on our findings, we propose the hypothesis that environmental pollutants may ‘mimic’ the impact of pathogen invasion on the immune system in autoimmune-susceptible individuals.

## Simple Summary

During an infection, foreign antigens that share similarities with ‘self’ proteins may trigger the immune system to attack healthy tissues, leading to autoimmune diseases. However, despite a decrease in infectious burden, autoimmune disease incidence is on the rise. To address this seeming paradox, we propose the hypothesis that environmental pollutants may ‘mimic’ the impact of pathogen invasion on the immune system in autoimmune-susceptible individuals.

## 1. Introduction

Autoimmune diseases are a group of chronic, debilitating diseases that feature the attack of self-tissues by the immune system [1]. While the exact cause of autoimmune diseases remains undefined, current hypotheses highlight a complex interaction between genetic susceptibility and environmental triggers such as infectious agents [1]. Many hypotheses on autoimmune etiology invoke a preceding infection as the initiating factor, which are thought to cause autoimmune reactions through molecular mimicry as discussed further below, epitope spreading (i.e., the diversification of epitope specificity), and bystander activation (i.e., the activation of T/B cells in an antigen-independent manner) [1,2,3,4,5].

Since the early description on the similarity between the *S. pyogenes* membrane and mammalian muscle in 1966, a number of studies have suggested that homology between pathogens and mammalian molecules may underlie autoimmune diseases, a theory termed ‘molecular mimicry’ [6]. This theory states that similarities between foreign and self-antigens can favor an activation of autoreactive T or B cells by foreign-derived antigens in a susceptible individual, inducing autoimmunity [6]. On the molecular level, molecular mimicry can happen given the polyspecificity of TCRs (T-cell receptors) or BCRs (B-cell receptors), i.e., the ability of TCR/BCR to recognize multiple distinct peptide/MHC ligands such as microbe and self-antigens) [7]. Additionally, some T cells exhibit double or chimeric TCRs, which allow them to recognize pathogen- or self-peptides depending on the scenario, evade central tolerance, and induce autoimmunity [6].

Molecular mimicry has been examined in many autoimmune diseases. Study of multiple sclerosis demonstrates the presence of T cells with specificity for both myelin basic protein (MBP), the characteristic autoantigen in the disease, and the EBV nuclear antigen 1 (EBVNA1) [8]. Rheumatoid arthritis (RA) is associated with infectious agents including *P. gengivalis*, *P. mirabilis*, *E. coli* and EBV, with multiple cross-activity for microbial and self-antigens detected [9,10]. Additionally, levels of antibodies against *P. mirabilis* urease F (UreF) in RA patients correlate positively with disease features such as rheumatoid factor and C-reactive protein levels [11]. Urinary tract infection is associated with increased risk of the primary biliary cirrhosis, and patients display antibodies that react against both human and *E. coli* PDC-E2 [12]. In systemic lupus erythematosus, there is higher incidence of EBV infection as well as higher titers of antibodies against EBV in patients and there is evidence that humoral response against EBVNA-1 stimulates the production of anti-dsDNA and anti-Sm antibodies in genetically susceptible individuals [13].

While molecular mimicry is recognized as one of the leading mechanisms by which infectious agents may induce autoimmunity [6], it does not directly explain the inverse relationship between the decrease in infectious burden and increase in autoimmune disease incidence observed in industrialized countries [14]. The objective of this study is to generate an initial hypothesis that may address this seeming paradox by probing into the relationship of environmental pollutant and pathogen sequences in an autoimmune model.

### 1.1. Relationship Between Pollutant, Pathogen, and Autoimmunity

#### 1.1.1. Study Model

To this end, we focused on the environmental pollutant benzo[a]pyrene (BaP), a polycyclic aromatic hydrocarbon commonly found in byproducts of industrial incineration, motor vehicle emissions, and cigarettes [15], and studied its effect on the autoimmune-prone mouse strain MRL. The MRL mice are genetically predisposed to autoimmunity and start to exhibit multi-organ, lupus-like disorders including glomerulonephritis, arthritis, skin rash, cerebritis, and anti-dsDNA antibodies at a late stage (~18 months; corresponding to ~56 years of age in humans) [16]. We exposed MRL mice to BaP at a young age (2 months old), before manifestation of lupus-like phenotypes, to understand the interaction between environmental agents and genetic factors in triggering autoimmunity.

#### 1.1.2. BaP Impact on MRL Spleen

**Figure 1:**
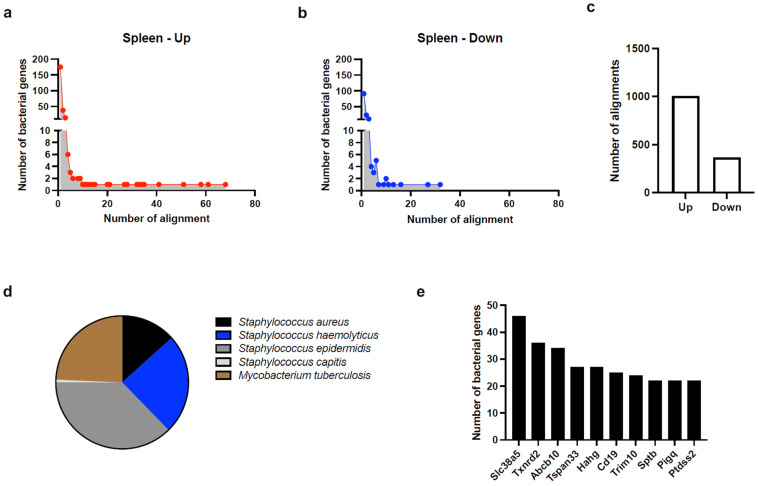
Alignment of BaP-regulated genes in the MRL spleen to bacterial genes. (**a**) number of bacterial genes aligned to each BaP-upregulated gene in the MRL spleen. (**b**) number of bacterial genes aligned to each BaP-downregulated gene in the MRL spleen. (**c**) number of alignments between bacterial genes and BaP-upregulated (Up) vs. BaP-downregulated (Down) genes. (**d**) number of alignments for each bacterial species. (**e**) top BaP-upregulated, MRL genes with similarities to bacterial genes. All nucleic acid alignments were confirmed on the protein levels.

BaP exposure caused significant changes in gene expression in organs including the spleen and kidney in the MRL mice, which was determined by RNA-Seq (q-value < 0.05) [17]. The spleen was analyzed because it is the main filter for blood-borne pathogens and antigens as well as a key organ regulating local and systemic immunity. The top 60 genes that were up- or downregulated, respectively, by BaP in the MRL spleen were examined to assess if they exhibited homology to components of any bacterial pathogens that are known to infect the spleen, on both the nucleic acid and protein levels. These pathogens, typically acquired through bacteremia, included *Mycobacterium tuberculosis*, *Staphylococcus aureus*, *Staphylococcus haemolyticus*, *Staphylococcus epidermidis*, and *Staphylococcus capitis*. An initial nucleic acid BLAST was performed between genes in these bacteria and BaP-regulated genes in the mouse spleen, and subsequently the similarities of hits were confirmed using protein alignment. As a standard method for sequence alignment, BLAST controls for random sequences and database sizes [18]. The BLAST yielded a total number of 1006 alignments between the bacterial proteins and the protein products of BaP-upregulated MRL genes (Figure 1a,c). In contrast, there were a total of 366 alignments between individual bacterial proteins and proteins coded by BaP-downregulated genes in MRL (Figure 1b,c). This corresponds to a 2.74-fold enrichment for BaP-upregulated proteins, as compared to downregulated ones, that show homology with bacterial components. Most alignments were one-to-one, with 175 alignments to BaP-upregulated and 91 to BaP-downregulated sequences, respectively. However, there were some exceptions, such as *wag22* and *pknH* from *M. tuberculosis*, which exhibited more than ten alignments with MRL sequences upregulated by BaP. As additional controls, housekeeping genes in the MRL mice were not aligned to bacterial genes under the same setting.

*S. epidermidis* showed the greatest number of genes aligned to BaP-stimulated genes in the MRL spleen, with 120 genes with homology to the host, corresponding to 4.98% of its protein-coding sequences (Figure 1d). This is followed by *M. tuberculosis*, with 78 genes showing homology to BaP-upregulated spleen genes (1.91% of its protein-coding sequences), and *S. haemolyticus*, with 78 genes showing homology to BaP-upregulated spleen genes (0.79% of its protein-coding sequences). On the host side, several BaP-stimulated genes showed homology with more than thirty bacterial genes examined, including *Slc38a5* (Solute Carrier Family 38 Member 5), *Txnrd2* (Thioredoxin Reductase 2) and *Abcb10* (ATP Binding Cassette Subfamily B Member 10) (Figure 1e).

#### 1.1.3. BaP Impact on MRL Kidney

**Figure 2:**
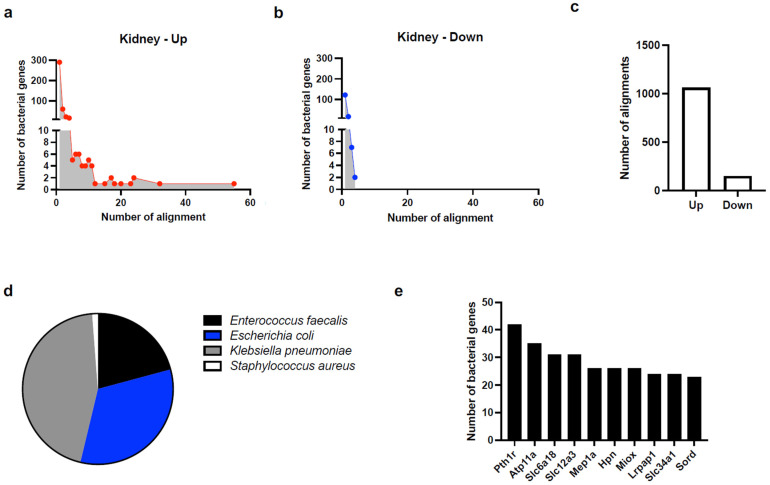
Alignment of BaP-regulated genes in the MRL kidneys to bacterial genes. (**a**) number of bacterial genes aligned to each BaP-upregulated gene in the MRL kidneys. (**b**) number of bacterial genes aligned to each BaP-downregulated gene in the MRL kidneys. (**c**) number of alignments between bacterial genes and BaP-upregulated (Up) vs. BaP-downregulated (Down) genes. (**d**) number of alignments for each bacterial species. (**e**) top BaP-upregulated, MRL genes with similarities to bacterial genes. All nucleic acid alignments were confirmed on the protein levels.

In addition to spleen, the kidney was examined because lupus nephritis is among the most common and severe manifestations of systemic lupus erythematosus [19]. Similarly, we asked if BaP-regulated genes in the kidney (q < 0.05) showed similarity to components of bacterial pathogens known to infect the kidney, on both the nucleic acid and protein levels. These pathogens included *Enterococcus faecalis*, *Escherichia coli*, *Klebsiella pneumoniae*, and *Staphylococcus aureus.* An initial DNA BLAST was performed, followed by confirmatory BLAST on the protein level, which yielded a total number of 1062 alignments (on both the DNA and protein levels) between bacterial and BaP-regulated genes in the MRL kidney (Figure 2a,c). In contrast, there was a total of 108 alignments between bacterial and MRL genes downregulated by BaP (Figure 2b,c). This corresponds to a 9.83-fold enrichment for BaP-upregulated genes showing homology with bacterial genes. Similarly, most bacterial genes had one alignment to MRL genes, with exceptions of multiple alignments especially seen in BaP-upregulated kidney genes (Figure 1a).

*K. pneumoniae* showed 186 genes with homology to BaP-upregulated genes in the MRL kidney, corresponding to 3.38% of its protein-coding sequences (Figure 2d). This was followed by *E. coli*, with 136 genes aligned to BaP-upregulated spleen genes (3.23% of its protein-coding sequences), and *E. faecalis*, with 86 genes aligned to BaP-upregulated spleen genes (2.97% of its protein-coding sequences). In comparison, *S. aureus* showed limited alignment to BaP-stimulated mouse genes (5 genes). In the host, top BaP-stimulated genes that aligned with more than thirty bacterial genes included *Pth1r* (Parathyroid Hormone 1 Receptor), *Atp11a* (ATPase Phospholipid Transporting 11A), *Slc6a18* (Solute Carrier Family 6 Member 18), and *Slc12a3* (Solute Carrier Family 12 Member 3) (Figure 2e).

#### 1.1.4. The Hypothesis of Pollution-Triggered Molecular Mimicry

Our analysis showed that BaP, one of the most studied polycyclic aromatic hydrocarbons (PAHs), stimulated the expression of genes with homology to bacterial components in an autoimmune-prone mouse model. There was an enrichment of proteins expressed from BaP-upregulated genes, as compared to BaP-downregulated genes, that showed homology to potential bacterial antigens. Combined with additional pre-autoimmune stimuli such as proinflammatory cell death as well as genetic polymorphisms altering the polyspecificity of TCR/BCRs, peptides expressed from these genes may be displayed to the immune system in a way that ultimately facilitate a hyperactivated immune reaction. It is hypothesized that environmental pollutants may initiate this type of ‘sterile’ molecular mimicry in autoimmune pathogenesis.

The major bacterial species with similarity to BaP-regulated proteome include *K. pneumoniae*, which has been suggested to trigger autoimmune diseases such as ankylosing spondylitis through its homology to human proteins including HLA-B27 [20]. Similarly, *M. tuberculosis* components may initiate or exacerbate systemic lupus erythematosus via structural homology [21].

Top host proteins that may resemble bacterial antigens under BaP stimulation feature transporters such as *Slc38a5*, *Slc6a18*, *Slc12a3*, *Abcb10*, and *Atp11a.* Of note, the thiazide-sensitive sodium-chloride cotransporter *Slc12a3* is a direct target of autoantibodies in autoimmune diseases such as Sjögren’s syndrome, autoimmune thyroiditis and scleroderma [22]. Furthermore, several Slc family members have been linked to inflammatory conditions [23], prompting further study examining the role of transporters and metabolism in autoimmunity.

## 2. Conclusions

Based on our initial study, we propose the hypothesis that environmental pollutants ‘mimic’ pathogen evasion study to induce autoimmunity. Our analysis supports previous findings that autoimmune diseases including multiple sclerosis, rheumatoid arthritis, and systemic lupus erythematosus associate with infectious agents [8,9,10,11,12,13]. Of note, in 2011, a new syndrome termed ‘ASIA Autoimmune/Inflammatory Syndrome Induced by Adjuvants’ was defined, which summarizes the spectrum of immune-mediated diseases triggered by an adjuvant stimulus such as chronic exposure to silicone, tetramethylpentadecane, pristane and other adjuvants [24]. These environmental factors have been found to induce autoimmunity, pointing to the interplay between a genetically susceptible background and exposure-induced autoimmunity. Our proposed hypothesis fits in this diagram of genetic–environmental interaction in autoimmune pathogenesis and further suggests the induction of microbial-associated sequences as one of the possible mechanisms in the induction of autoimmunity.

As a hypothesis article, this report is limited in experimental validation such as MHC-peptide binding and validation in human populations. Future studies should be designed to test the ‘pollution-triggered molecular mimicry hypothesis’ proposed in this article.

In contrast to the decrease in infectious burden in many areas around the world, PAHs (polycyclic aromatic hydrocarbons) bound to PM2.5 have increased especially in regions with rapid economic expansion and increased traffic [25]. Furthermore, there is significant uptake of BaP from such food as grilled meats, processed vegetable oils and crops grown in contaminated environment [26]. BaP wildfire emissions increasingly impact global air quality [27]. Together with our finding that BaP increases the expression of genes that resemble microbial components on the nucleic acid and protein levels, it is proposed that environmental pollution may interact with microbial imbalance to challenge the immune system and contribute to increased autoimmune disease incidence worldwide.

## 3. Methods

### 3.1. Gene Expression in BaP-Treated Mice

Differentially expressed genes were identified in previous reported studies on MRL mice treated with BaP [17]. Briefly, tissue samples were collected from MRL mice treated with Benzo(a)pyrene (BaP) or vehicle over the course of 8 weeks following established procedures [17]. Tissues were subjected to spatial transcriptomics following the Visium tissue preparation guidelines. Sequencing libraries were sequenced on the NovaSeq sequencer, and differential gene expression analysis was performed using the Space Ranger pipeline.

### 3.2. Identification of Pathogen of Interest

To explore potential similarities between BaP-responsive mouse genes and bacterial genes, the literature on bacterial pathogenicity and virulence, specifically targeting pathogens known to infect the spleen and kidneys, was reviewed. Supplementary data were obtained from the NIH Pathogen Database and related publication, based on which a curated list of relevant bacterial species was compiled for comparative genetic analysis. Pathogens of interest were determined by clinical relevance, number of reported cases in the US and impact on the immune system of the host.

### 3.3. Sequence Alignment

BLAST (Basic Local Alignment Search Tool, version BLAST + 2.17.0) was utilized to align mouse genes with bacterial sequences and determine the degree of nucleotide similarity, following standard, published procedures using e-values (0.01) as significance cutoff [18]. For genes with significant alignment on the nucleotide level, their protein sequences were obtained from UniProt [28] and homology on the protein level was confirmed using ClustalOmega 1.0 [29].

## Data Availability

No new data was generated in this study. Raw data has been published [17] and is available upon request.
